# Early detection of pine wilt disease tree candidates using time-series of spectral signatures

**DOI:** 10.3389/fpls.2022.1000093

**Published:** 2022-10-13

**Authors:** Run Yu, Langning Huo, Huaguo Huang, Yuan Yuan, Bingtao Gao, Yujie Liu, Linfeng Yu, Haonan Li, Liyuan Yang, Lili Ren, Youqing Luo

**Affiliations:** ^1^ Beijing Key Laboratory for Forest Pest Control, Beijing Forestry University, Beijing, China; ^2^ Department of Forest Resource Management, Swedish University of Agriculture Sciences, Umeå, Sweden; ^3^ Research Center of Forest Management Engineering of State Forestry and Grassland Administration, Beijing Forestry University, Beijing, China; ^4^ French National Research Institute for Agriculture, Food and Environment (INRAE)—Zoologie Forestiere Centre de recherche d’Orléans, Orléans, France; ^5^ Sino-French Joint Laboratory for Invasive Forest Pests in Eurasia, Beijing Forestry University—French National Research Institute for Agriculture, Food and Environment (INRAE), Beijing, China

**Keywords:** pine wilt disease, unmanned aerial vehicle, hyperspectral images, multi-temporal data, remote sensing, early detection, machine learning

## Abstract

Pine wilt disease (PWD), caused by pine wood nematode (PWN), poses a tremendous threat to global pine forests because it can result in rapid and widespread infestations within months, leading to large-scale tree mortality. Therefore, the implementation of preventive measures relies on early detection of PWD. Unmanned aerial vehicle (UAV)-based hyperspectral images (HSI) can detect tree-level changes and are thus an effective tool for forest change detection. However, previous studies mainly used single-date UAV-based HSI data, which could not monitor the temporal changes of disease distribution and determine the optimal detection period. To achieve these purposes, multi-temporal data is required. In this study, *Pinus koraiensis* stands were surveyed in the field from May to October during an outbreak of PWD. Concurrently, multi-temporal UAV-based red, green, and blue bands (RGB) and HSI data were also obtained. During the survey, 59 trees were confirmed to be infested with PWD, and 59 non-infested trees were used as control. Spectral features of each tree crown, such as spectral reflectance, first and second-order spectral derivatives, and vegetation indices (VIs), were analyzed to identify those useful for early monitoring of PWD. The Random Forest (RF) classification algorithm was used to examine the separability between the two groups of trees (control and infested trees). The results showed that: (1) the responses of the tree crown spectral features to PWD infestation could be detected before symptoms were noticeable in RGB data and field surveys; (2) the spectral derivatives were the most discriminable variables, followed by spectral reflectance and VIs; (3) based on the HSI data from July to October, the two groups of trees were successfully separated using the RF classifier, with an overall classification accuracy of 0.75–0.95. Our results illustrate the potential of UAV-based HSI for PWD early monitoring.

## Introduction

**Figure 1:**
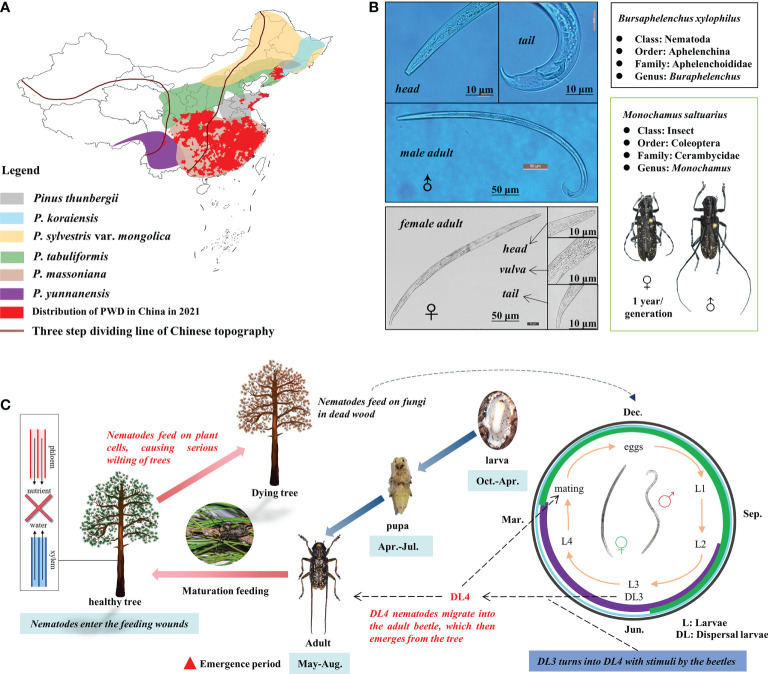
Distribution of pine wilt disease in China in 2021 **(A)**, morphology of *Monochamus saltuarius* and *Bursaphelenchus xylophilus* **(B)**, and pathogenic mechanism of pine wilt disease (PWD) **(C)**.

The pine wood nematode (*Bursaphelenchus xylophilus*; **Figure 1B**) is a dangerous invasive species that causes pine wilt disease (PWD), which has already destroyed enormous areas of pine forest in China (**Figure 1A**). In natural conditions, *B. xylophilus* spreads through vector insects (*Monochamus saltuarius* in Northeast China; **Figure 1B**). *B. xylophilus* invades through the wounds caused by vector insects feeding on pine trees and eats the xylem, resulting in the wilting and death of a pine tree due to the obstruction of water transport (**Figure 1C**) (Mamiya, 1983; Kobayashi et al., 2003). Pine trees infested with PWD die rapidly within three months (Shin, 2008; Yu et al., 2021c). Therefore, early-stage PWD detection is crucial for a prompt management response. Early detection requires advanced and effective methods like remote sensing (RS).

Unmanned aerial vehicle (UAV) RS is a powerful technology for detecting forest pests and diseases (Syifa et al., 2020; Wu et al., 2021; Xia et al., 2021). UAVs mounted with visible-light or multispectral sensors can rapidly collect high-resolution images of forest crowns with high flexibility. However, using only visible-light or multispectral data at the early stages of a *B. xylophilus* infestation leads to low infestation detection accuracy (Wu et al., 2021; Yu et al., 2021b). There are two main reasons for this low performance: (1) The spectral information is insufficient, and (2) the width of multispectral bands is too wide to be successfully applied for early detection (Yang et al., 2017; Abdullah et al., 2019b).

Hyperspectral RS uses data from hundreds of bands and continuous wavelengths, and these bands can capture physiological changes in infested trees, which help detect early-stage pest and diseases infestations (Cheng et al., 2010; Abdullah et al., 2018; Liu et al., 2021; Yu et al., 2021d). As the advanced technology used in forest pest and diseases detection, UAV-based hyperspectral imagery (HSI) can provide highly accurate detection with flexible and efficient data acquisition (Iordache et al., 2020; Li et al., 2020; Zhang et al., 2018; Lin et al., 2019; Lin et al., 2021; Yu et al., 2021a). Most studies were based on single-date UAV-based hyperspectral data for early monitoring of forest pest and diseases (Lausch et al., 2013; Li et al., 2020; Zhang et al., 2018; Lin et al., 2019; Lin et al., 2021; Yu et al., 2021a; Yu et al., 2021c; Yu et al., 2021d). Compared with single-date data, the use of time-series data is relatively rare (Iordache et al., 2020; Einzmann et al., 2021; Bárta et al., 2022), even though it could be used to capture more complete infestation processes and determine the optimal detection periods, thus leading to more reliable results than those based on a single date, with the limitation that any changes observed in the color and texture of tree crowns could also be due to phenology and other factors. In a PWD study, Iordache et al. (2020) acquired UAV-based HSI data in June 2018, October 2018, June 2019, September 2019 and October 2019. Even though data were obtained for two years, the collection interval was too long to capture early subtle changes in tree canopies. In addition, while the emergence of vector insects (*Monochamus galloprovincialis* in his study) began in May, data collection did not begin until June, which may have been too late.

In our study area, the flight period of the vector insect *M. saltuarius* is from May to October, producing one generation a year. Therefore, six UAV-based HSI data acquisitions in the study area were performed from May to October 2021. Using these multi-temporal data, we explored the following questions: (1) Which are the most discriminable spectral features? (2) When are variations in the spectral features due to PWD first detectable?

## Material and methods

**Figure 2:**
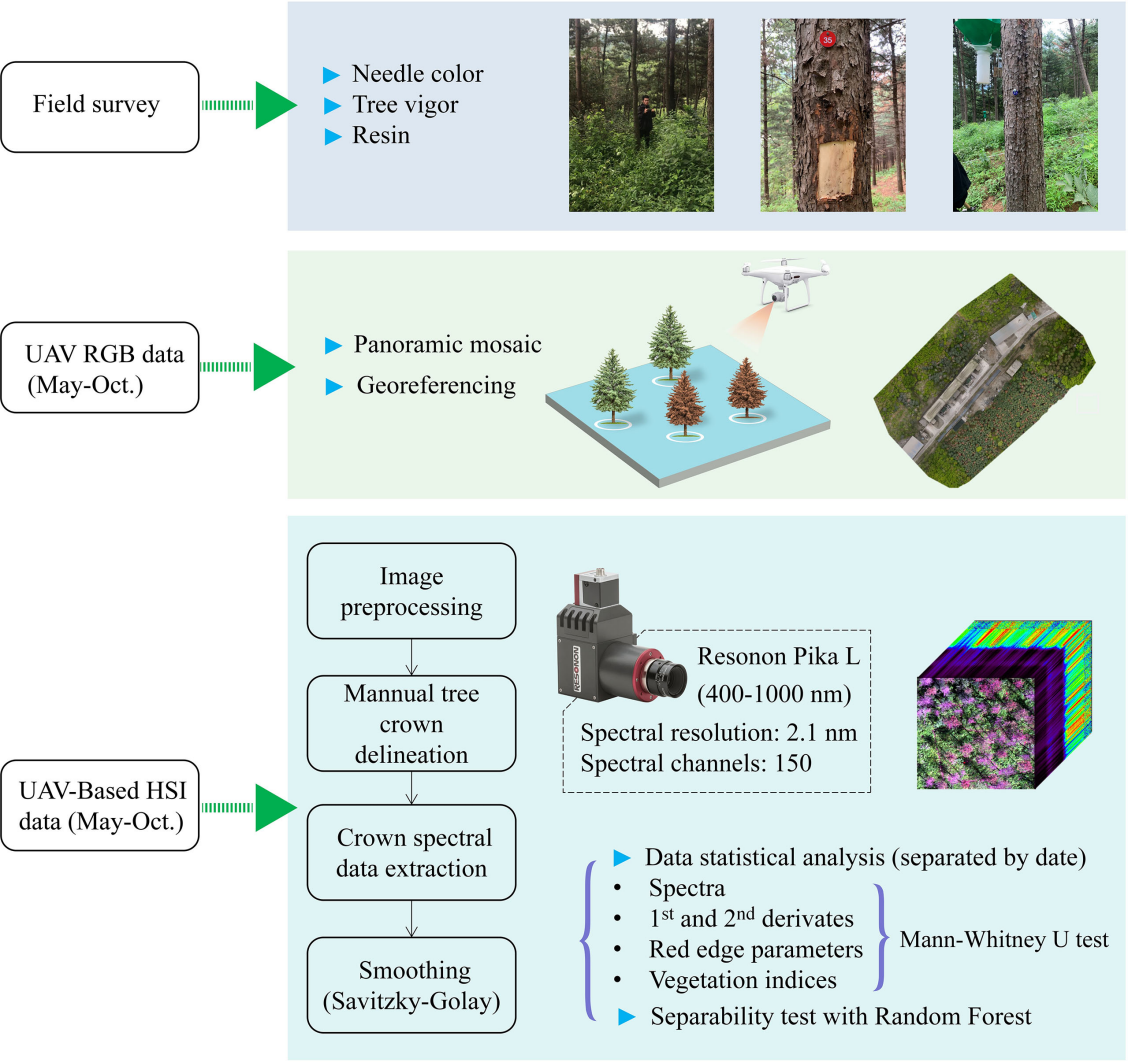
Flowchart of data acquisition and processing.

The study involved field survey, UAV-based RGB data and HSI data (**Figure 2**). The study site and the methods employed for data analysis are described in the next sub-sections.

### Study sites and field survey

**Figure 3:**
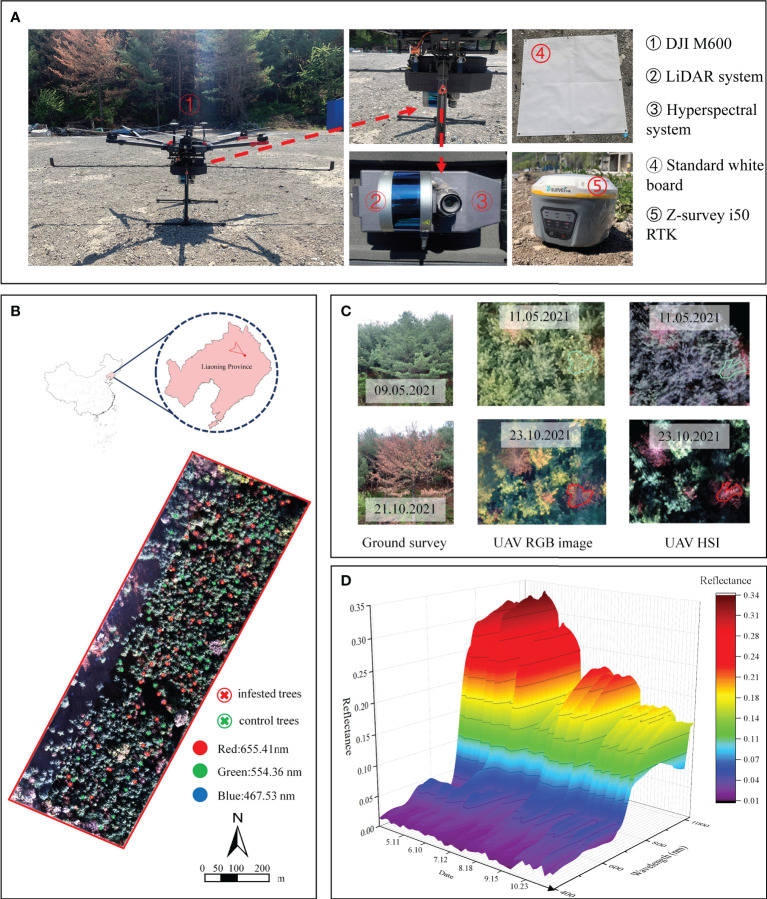
The UAV-based hyperspectral and LiDAR system **(A)** and location of the study area and the hyperspectral image of the test site acquired on 23 October **(B)**. An example pine tree shows the process of discoloration in the field, UAV RGB and hyperspectral images **(C)** and examples of hyperspectral curves of sample trees at different dates **(D)**.

The study area is located in Dongzhou District, Fushun City, Liaoning Province, northeast China (124°12′36″–124°13′48″ E, 41°56′53″–41°57′46″ N; **Figure 3A**). Plantation forests in the study area are dominated by *Pinus koraiensis*, aged approximately 40–50 years.

Field surveys were carried out six times: on 9 May, 9 June, 11 July, 11 August, 13 September, and 21 October 2021. On 9 May, 178 trees with no discoloration were selected from the study area, and whether each tree carried the *B. xylophilus* was verified through morphological and molecular identification. The results showed that 59 trees were confirmed to be infected with the *B. xylophilus*. The same number of healthy trees were selected as control. In the remaining five surveys, resin secretion (observed by making wounds on trees), tree vigor, and needle color of pine trees were recorded in each plot. A handheld differential global positioning system (DGPS, Version S760) was applied to measure the location of each sampled tree with sub-meter accuracy.

### UAV-based data acquisition and preprocessing

A DJI Matrice 600 Pro UAV (DJI, Shenzhen, Guangdong, China) equipped with a Pika L hyperspectral camera (Resonon, Bozeman, MA, USA; **Figure 3A**) and a LR1601-IRIS LiDAR system (IRIS Inc., Beijing, China; **Figure 3A**) were used to acquire HSI and LiDAR (light detection and ranging) data from 11:50–12:30, on 11 May, 10 June, 12 July, 18 August, 15 September, 23 October 2021 under sunny and cloudless weather conditions. The main parameters of Pika L hyperspectral system are shown in **Table 1**. LR1601-IRIS LiDAR system is uncalibrated, and the pulse repetition frequency, laser wavelength, and returns per pulse are 5–20 Hz, 905 nm, and 2, respectively. An inertial measurement unit mounted on the UAV helped produce high-quality HSI data. Additionally, Z-survey i50 RTK (Shanghai Huace Navigation Technology Ltd., Shanghai, China) was used to improve the POS (position and orientation system) accuracy. HSI images of 4 cm/pixel ground sample distance (GSD, the distance between pixel centers measured on the ground) was obtained with a flight height of 120 m. The flight speed was 3 m/s, and the overlap was set to 60%. A standard board was set up in the flighting area, which was placed on a flat ground 3 meters near the boundary of our test forest and measured during each flight campaign. The standard board covered with PTFE material (poly tetra fluoroethylene), which is a Lambertian reflector, for correction and calibration of the HSI data. The reflectance of standard board was known (0.24-0.28 in our study). The DN value obtained by the hyperspectral camera was converted into the radiance value through the radiance calibration file, and then divided by the reflectances of the standard board in each band to obtain the total radiance. The reflectances of a target was obtained by dividing its radiance by the total radiance. The spectral range of the HSI was from 400 nm to 1000 nm with 150 spectral channels (spectral resolution of 3.3 nm). The whole UAV-based system is displayed in **Figure 3**. UAV RGB images were collected synchronously using a DJI Phantom 4 Pro (DJI, Shenzhen, Guangdong, China) under the same conditions. The RGB sensor was a 1-inch CMOS with a focal length of 24 mm. The flight altitude and speed were 120 meters and 3 m/s, respectively, and the GSD was 2.2 cm/pixel.

**Table 1:** Main parameters of the Pika L hyperspectral sensor.

Parameters	Values	Parameters	Values
Field of view	17.6°	Wavelength range	400-1000 nm
Focal length	17 mm	Spectral resolution	3.30 nm

After each data collection, the data was preprocessed using well-established processing routines (Holzwarth et al., 2011; Einzmann et al., 2021; Yu et al., 2021a), leading to top-of-canopy spectral bi-directional reflectances. The irradiance calibration, reflectance correction, and image mosaicking were conducted using the 3 m^2^ standard board and Spectronon software (Resonon, Bozeman, MA, USA), Megacube (LICA United Technology Limited, Beijing, China), ArcGIS (ESRI, Redlands, CA, USA), IDL 8.5 and ENVI 5.3 (Harris Corporation, Melbourne, FL, USA). The image geometric correction was conducted by using six ground control points, the location of which was measured using a DGPS device with an accuracy of sub-meter. The collected LiDAR data provided accurate DEM (digital elevation model) data for the HSI data preprocessing. The WGS1984 datum was applied as the coordinate system, and the LiDAR data were georeferenced in the Universal Transverse Mercator 51N. The ground, above-ground, and understory points were classified from the raw LiDAR data for HSI data preprocessing using the LiDAR360 software (version 4.1, GreenValley Inc., Beijing, China).

### Extraction of tree crowns

Tree crowns were extracted from the HSI of each date in ENVI 5.3 by manually drawing the ROIs (regions of interest) based on field survey, GPS measurements and RGB images. To minimize shadow influences, we only depicted the sunlit parts of the tree crown of all selected trees in all HSI datasets (**Figure 3C**). Mean sunlit crown reflectance spectra of 59 infested and 59 control tree crowns were extracted for further analysis. The same works of tree crowns extraction were conducted for the RGB data.

### Feature extraction

The raw spectral curves were smoothed with a Savitzky-Golay filter with 9 points wide sliding window and a second-order polynomial to reduce noises, which was performed in Origin 2021b software (OriginLab, Northampton, MA, USA). After smoothing the curves, first and second-order derivatives were calculated from the HSI data.

To further characterize different vegetation characteristics (e.g., chlorophyll content, cell structure, and water content), 13 vegetation indices (VIs, including leaf pigment indices, water stress indices, and red edge parameters) were computed for the smoothed spectral data (**Table 2**). For RGB data, the digital number values (DN) of the red, green, and blue bands of tree crowns were extracted for further analysis.

**Table 2:** Vegetation indices employed in the study.

Index	Description	Formula	Reference
**Leaf pigment indices**			
GI	Greenness Index	$\frac{R_{554}}{R_{677}}$	Smith et al. (1995)
NDVI	Normalized difference vegetation index	$\;\frac{R_{800}\;-\;R_{680}}{R_{800}\;-\;R_{680}}$	Rouse et al. (1974)
PRI	Photochemical Reflectance Index	$\;\frac{R_{570}\;-\;R_{531}}{R_{570}\;+\;R_{531}}$	Carter and Miller (1994)
PSRI	Plant Senescence Reflectance Index	$\;\frac{R_{677}\;-\;R_{500}}{R_{750}}$	Merzlyak et al. (1999)
PSSR	Pigment Specific Simple Ratio	$\frac{R_{800}}{R_{635}}$	Penuelas et al. (1997)
PSI	Plant Stress Index	$\frac{R_{695}}{R_{760}}$	Hunt and Rock (1989)
RVSI	Ratio Vegetation Stress Index	$\frac{R_{600}}{R_{760}}$	Hunt and Rock (1989)
RES	Red Edge Symmetry	$\;\frac{R_{718}\;-\;R_{675}}{R_{755}\;-\;R_{675}}$	Ju et al. (2010)
RENDVI	Red Edge Normalized Difference Vegetation Index	$\;\frac{R_{750}\;-\;R_{705}}{R_{750}\;+\;R_{705}}$	Gitelson and Merzlyak (1994)
**Water stress indices**			
WI1	Water Index	$\frac{R_{970}}{R_{900}}$	Penuelas et al. (1993)
WI2	Water Index	$\frac{R_{950}}{R_{900}}$	Hardisky et al. (1983)
**Red edge parameters**			
REIP	Red Edge Inflection Point	$700\;+\;40\;\times \;\frac{(R_{670}\;+\;R_{780})\;/\;2\;-\;R_{700}}{R_{740}\;-\;R_{700}}$	Danson and Plummer (1995)
REP	Wavelength position of the maximum first derivative of reflectance between 680 and 760 nm	–	Dawson and Curran (1998)

R_i,_ Reflectance at wavelength i.

### Separability evaluation and classification

A Mann-Whitney test was used to compare the spectral differences between infested and control trees in the images acquired at different times and identify when the infested trees showed different spectra reflectances than the control ones during the infestation process, which was conducted in IBM SPSS Statistics 23 software (IBM, New York, NY, USA).

A Random Forest (RF) classifier (Breiman, 2001) was applied to assess the discrimination ability of infested and control trees in different seasons. Four RF models were established using hyperspectral data, respectively using (1) derivatives, (2) spectra reflectances, (3) VIs and (4) all the above features. One RF model was built using the pixel values of the RGB image as a comparison to the hyperspectral data. All sample trees were employed for model training, and a 10-fold cross-validation was conducted. The 10-fold cross validation is to divide the training set into 10 sub samples, one single sub sample is retained as the data to validate the model, and the other 9 samples are used for training. The cross validation is repeated 10 times, and each sub sample is validated once, with an average of 10 times, and finally a single estimation is obtained (Waske et al., 2009). This method has been widely used in previous similar studies. (Lin et al., 2019; Yu et al., 2021c). The overall classification accuracy was calculated to assess the separability between infested and control trees. To show the features’ importance in the RF models, we also calculated the mean decrease accuracy (MDA) index of each feature. The higher the MDA value of a feature, the greater its importance (Abdel-Rahman et al., 2014; Huo et al., 2021). The RF classification was performed in the R software (Version 3.6.1) using the “randomForest” package. The models were trained with the default mtry (number of predictors randomly sampled for each node, set here as the square root of available input variables) and 1500 ntrees (the number of trees).

## Results

### Changes in PWD-infested trees assessed by field observations

**Figure 4:**
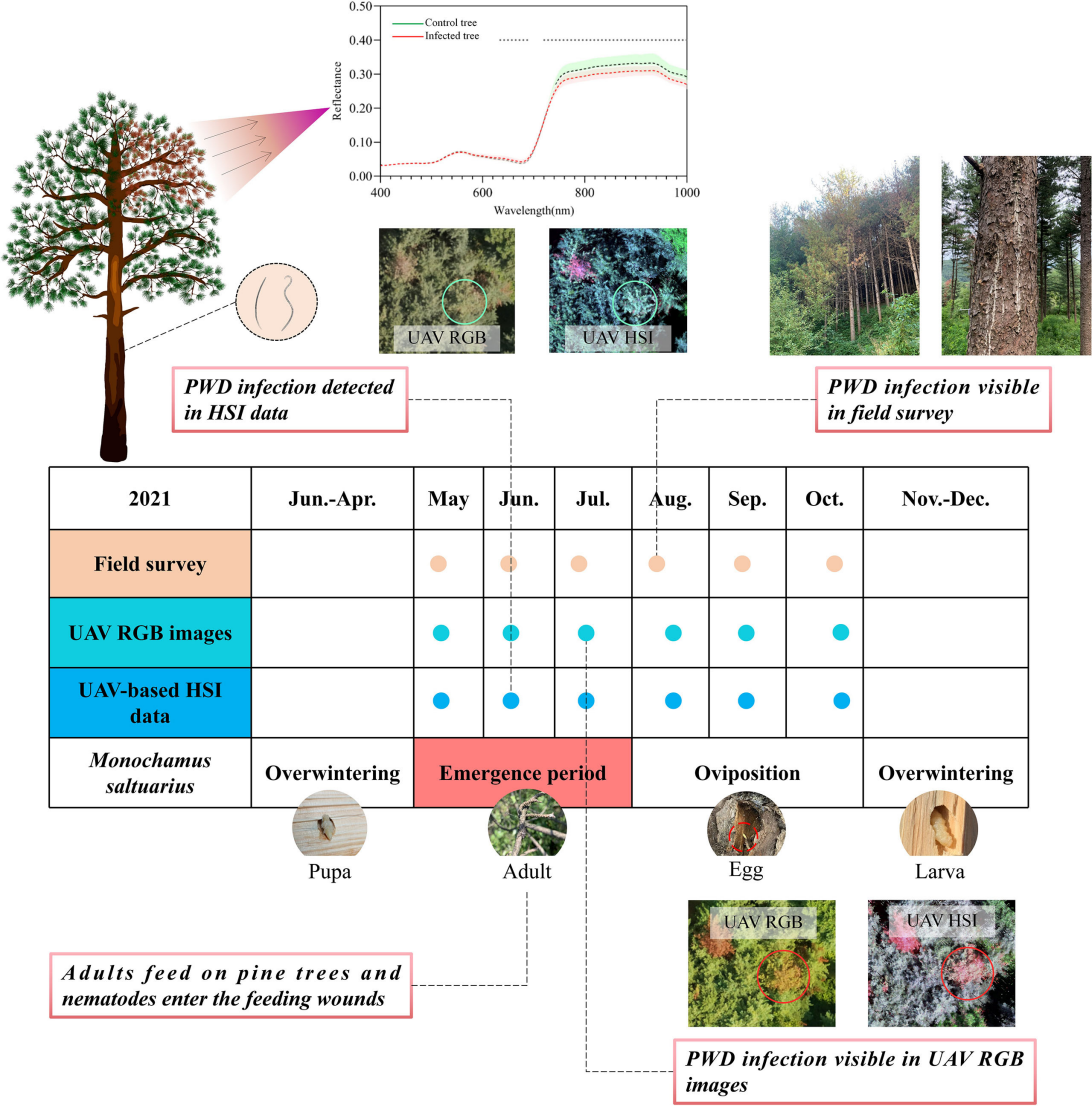
Overview of UAV-based RGB and hyperspectral data acquisitions and field surveys. The dots indicate data collection dates: field survey (pink), RGB images (blue-green), and hyperspectral data (blue).

Within the first three months (May to July), no evident changes in the infested pine trees were observed in the field investigations. In August, the color of the needles turned yellow and the resin secretion decreased in the infested trees (**Figure 4**). Resin secretion decreased with the increase of the degree of PWD infestation (Yu et al., 2021d). PWD-infested trees go from early-stage to late-stage infestation within five weeks (Umebayashi et al., 2017). During the field surveys, a few control trees also showed slight discoloration due to other factors. During September and October, the needles gradually turned reddish brown, and resin secretion further decreased in infested trees.

### Spectral-temporal changes of PWD infested trees

#### Hyperspectral reflectance and pixel values in the RGB images

**Figure 5:**
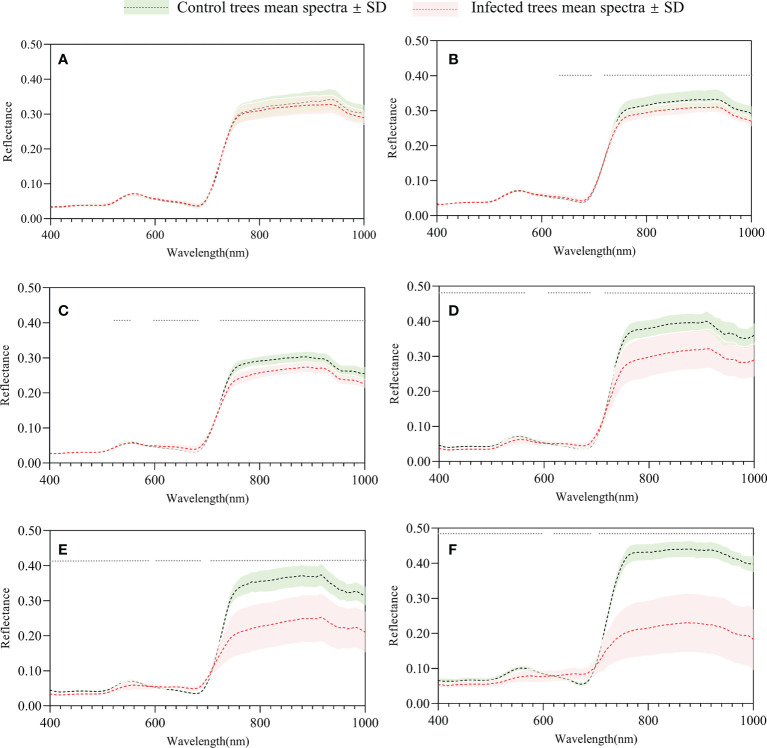
Mean spectra of infested and control trees of May **(A)**, June **(B)**, July **(C)**, August **(D)**, September **(E)**, and October **(F)**. The gray dots indicate a significant difference (p<0.01).

**Figure 6:**
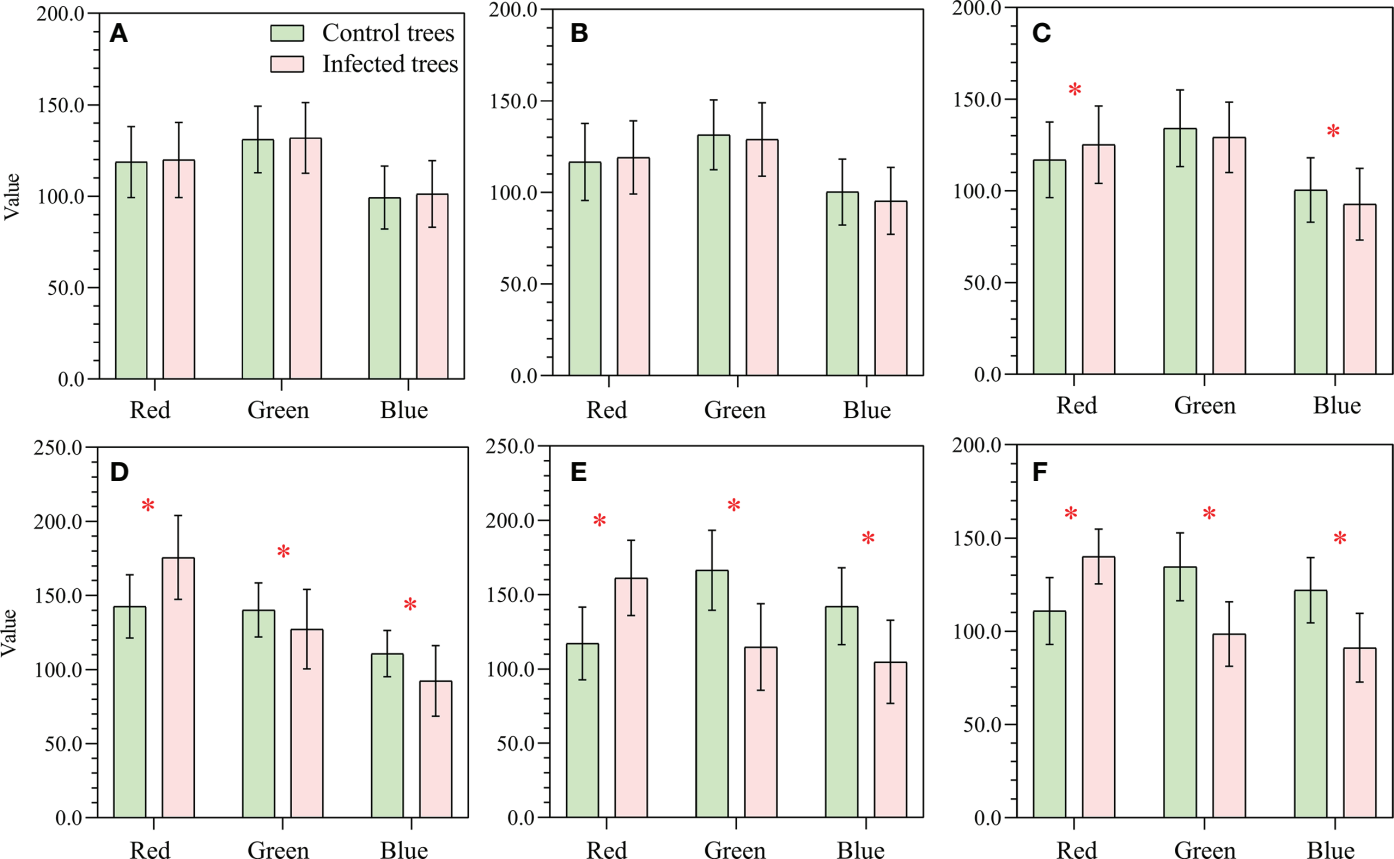
Mean RGB DN values (± standard deviation) for May **(A)**, June **(B)**, July **(C)**, August **(D)**, September **(E)**, and October **(F)**. The symbol * indicates a significant difference (p < 0.01).

The mean spectra of infested and control trees over time are shown in **Figure 5**. In May, the mean spectra of infested and control trees did not show significant difference (p<0.01)(**Figure 5A**). A change first appeared in June (p<0.01), and differences between the two groups were most obvious in October (**Figure 5F**). The reflectances declined in both groups in the green peak (490–580 nm) and near infrared (NIR; 780–1000 nm) and increased in the red edge (660–780 nm). This is caused by a decrease in chlorophyll content and a change in cell structure (Cheng et al., 2010; Meiforth et al., 2020). The mean pixel values in the RGB image (**Figure 6**) show that in the first two months, there was no significant difference between the two groups of trees, while a significant difference first appeared in the red and blue bands in July (p<0.01).

#### Derivatives, green peak and red edge parameters

**Figure 7:**
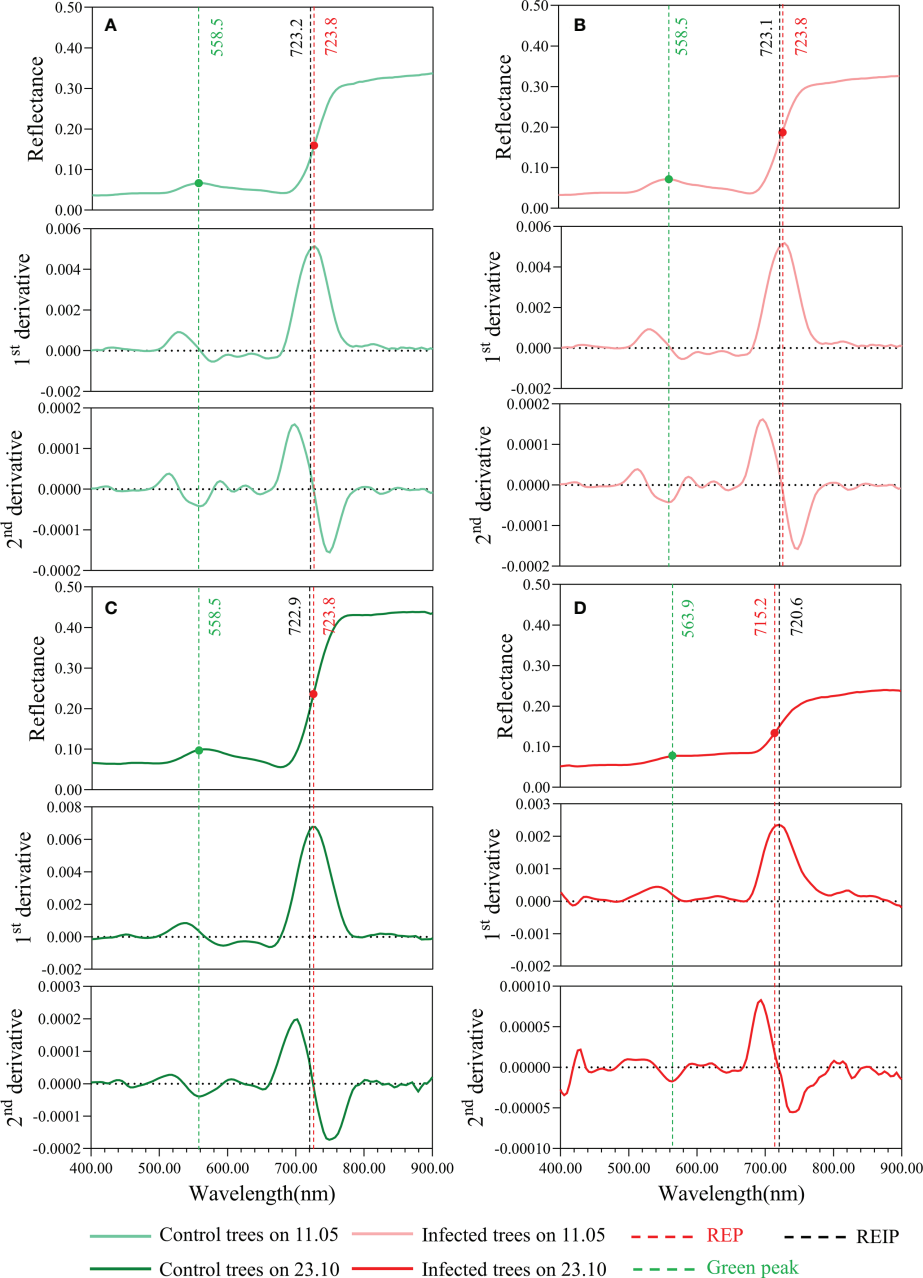
Mean tree crown spectra with 1^st^ and 2^nd^ derivative of infested trees (right) and control trees (left) on 11.05.2021 **(A, B)** and 23.10.2021 **(C, D)**. The green peak (green dots), red edge infestation point (black lines), and red edge position (red dots) are also displayed.

The 1^st^ and 2^nd^ derivatives and REPs (REP and REIP) are shown in **Figure 7**. A significant difference between the two groups was detected on the last date of HSI data acquisition. In the spectra of infested trees, the green peak shifted from 558.5 to 563.9 nm, while the REP moved from 723.8 to 715.2 nm. REIP shows similar trends to REP but with a smaller decrease (moved from 723.1 to 720.6 nm).

**Figure 8:**
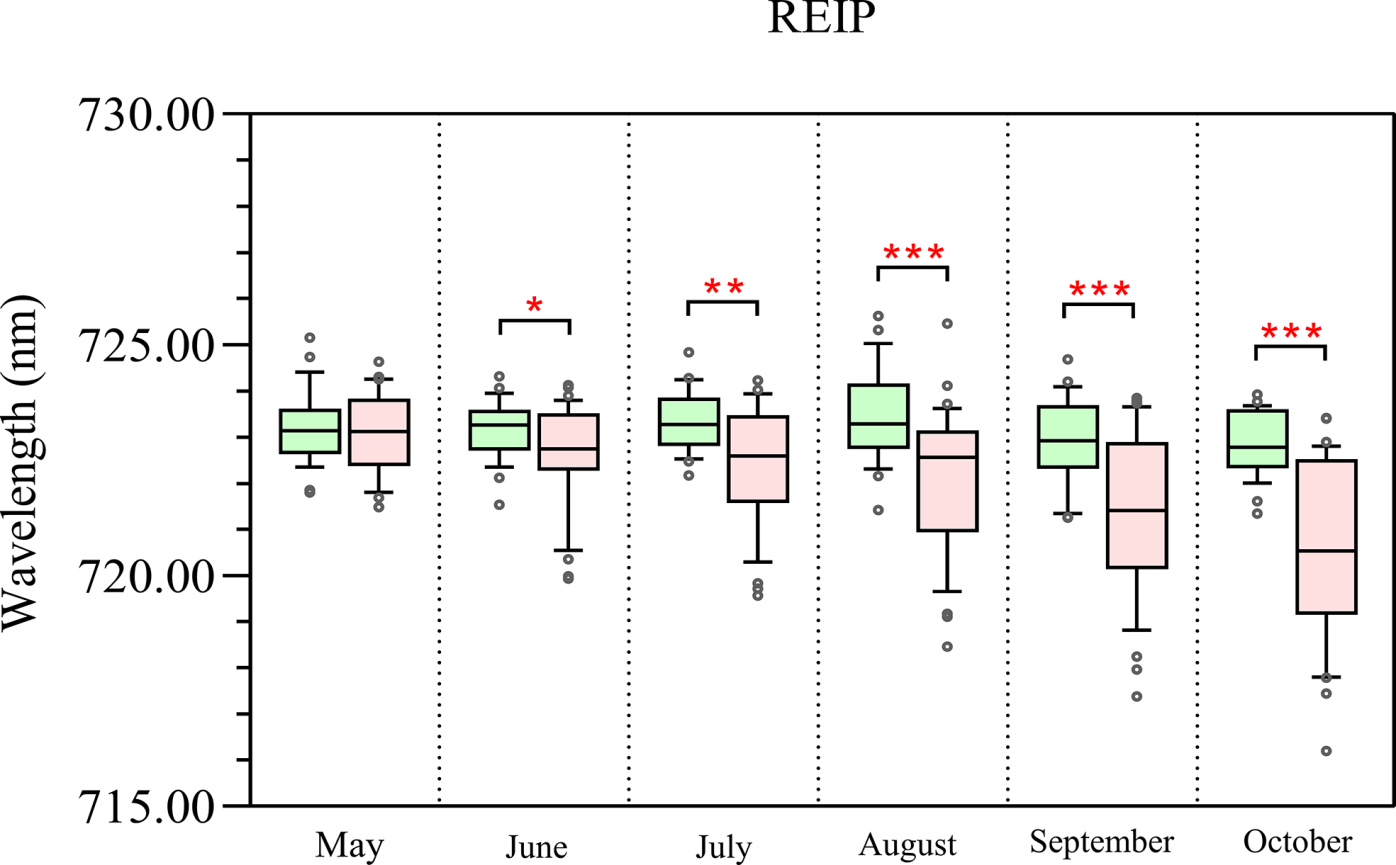
Temporal changes of the REIP derived from spectra of control (green) and infested trees (pink) over time. The symbol * indicates significant differences, with *, **, and *** indicating differences at p < 0.05, p < 0.01, and p < 0.001, respectively.

**Figure 8** shows the temporal development of REIP derived from tree canopy spectra of control and infested trees. In the first two sampling dates, the REIP values of the two groups were comparable, with only slight differences (p<0.05). The REIP values of the infested trees declined from June onwards (p<0.01 and p<0.001), while the control trees remained unchanged or fluctuated slightly (p<0.05).

#### Vegetation indices

**Figure 9:**
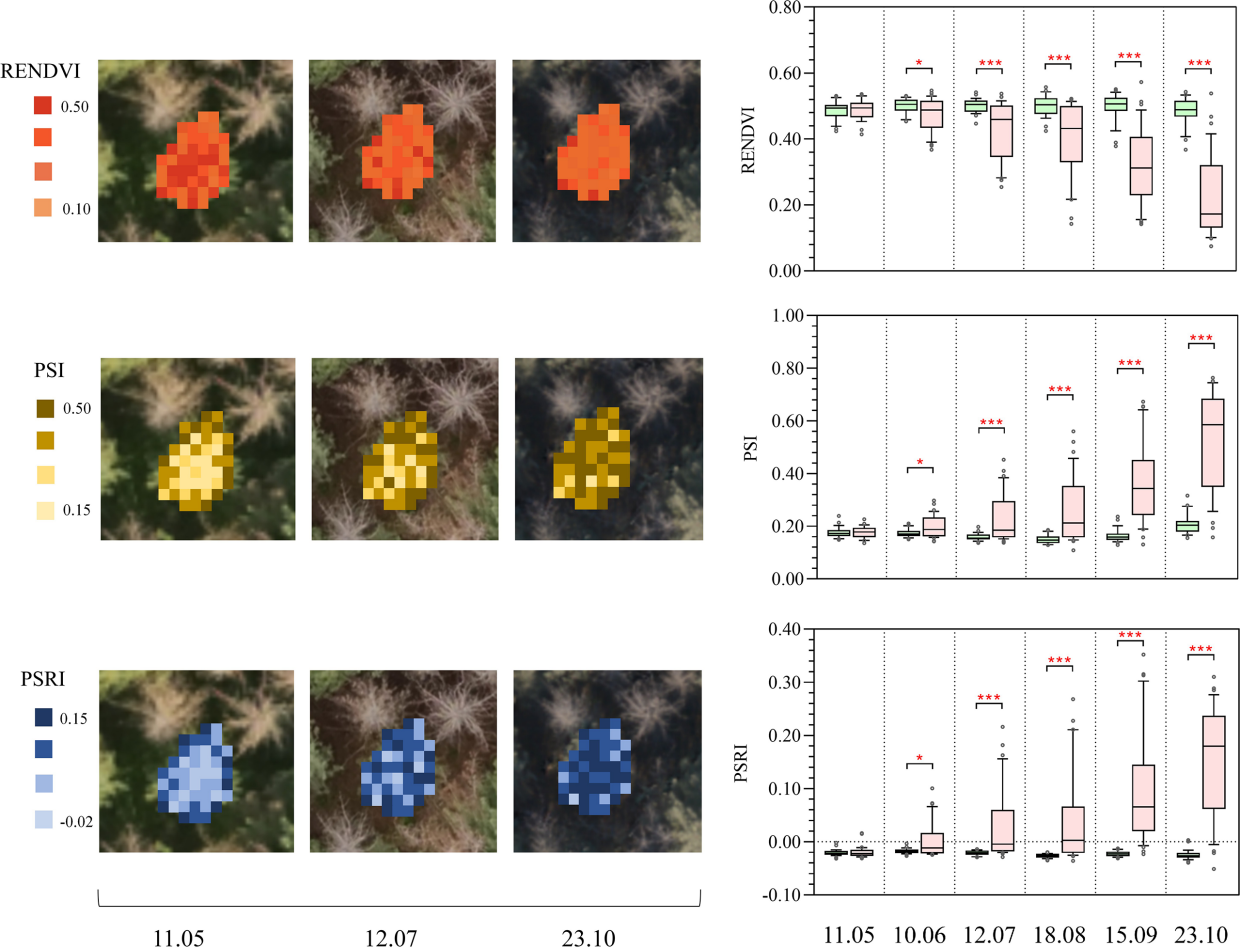
Rights: Boxplots showing temporal changes in Plant Senescence Reflectance Index (PSRI), Red Edge Normalized Difference Vegetation Index (RENDVI), and Plant Stress Index (PSI) derived from tree canopy spectra (infested trees are in pink, control trees are in green). The symbol * indicates significant differences, with * and *** indicating differences at p < 0.05, p < 0.01, and p < 0.001 levels, respectively. Left: associated maps of PSRI, RENDVI and PSI, showing the within-crown variation of features on 11 May, 12 July and 23 October.

The three most sensitive VIs (PSRI, RENDVI, and PSI) are shown as boxplots for two groups (**Figure 9**). From June onward, infested and control trees show significant differences in PSRI, RENDVI and PSI (p<0.01 and p<0.001). The PSRI and PSI values of infested trees increase from June to October, while RENDVI values gradually decrease. On the contrary, VIs values of control trees remain unchanged or change slightly (p<0.05).

### Random Forest classification and feature separability assessment

**Figure 10:**
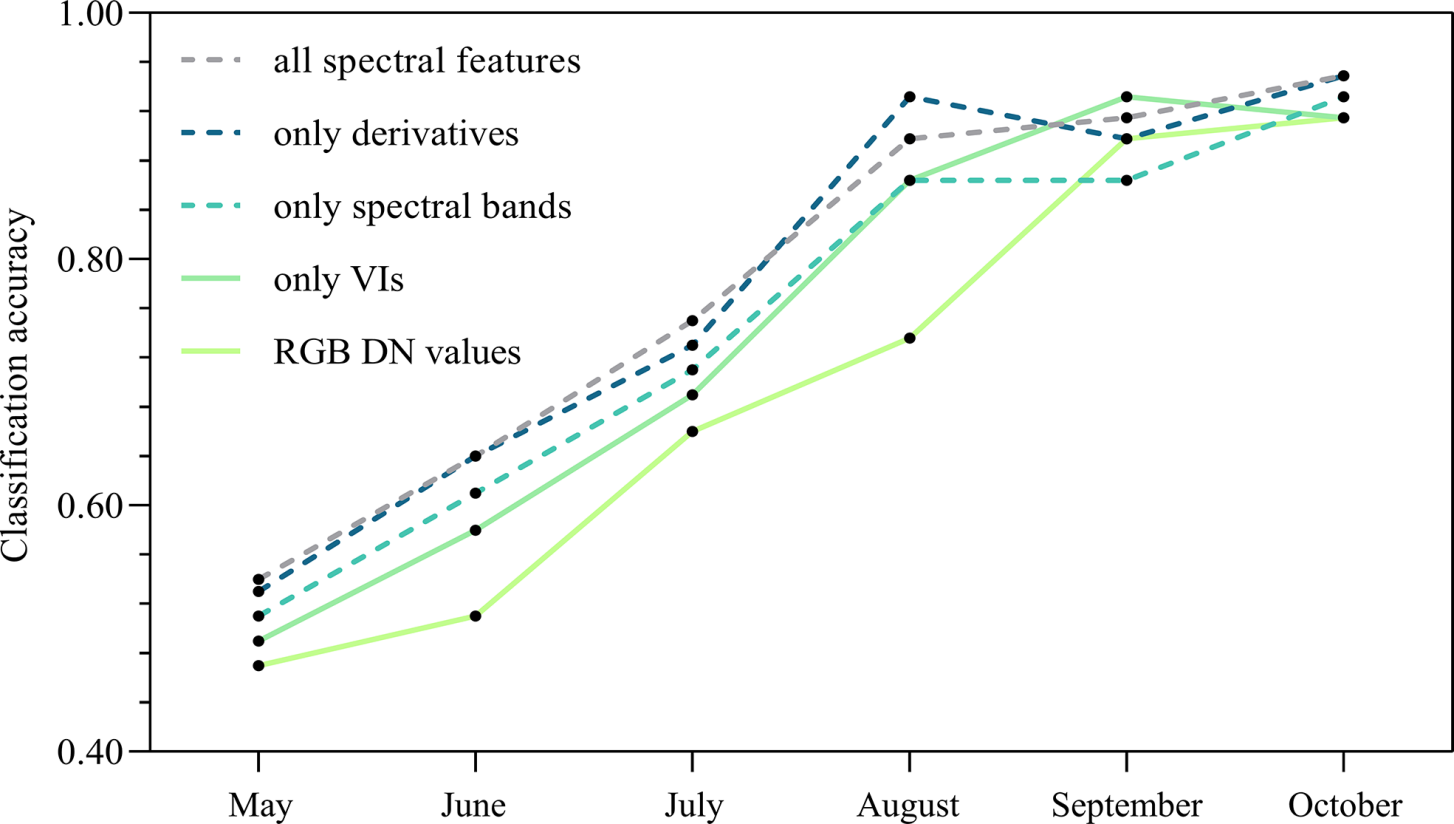
Classification accuracy for distinguishing infested and control trees by using different types of features during the course of pine wilt disease infestation.

All the features (spectral reflectance, derivatives, VIs and REPs) were employed in the RF classification model to separate infested and control trees. In the first two months, the two groups of trees could not be successfully discriminated (**Figure 10**). The two groups of trees were first separated in July with an overall accuracy of 0.75. The ability of distinguish the two groups was further improved over the remaining months, and the overall accuracy reached the highest (0.95) in the last sampling date.

**Figure 11:**
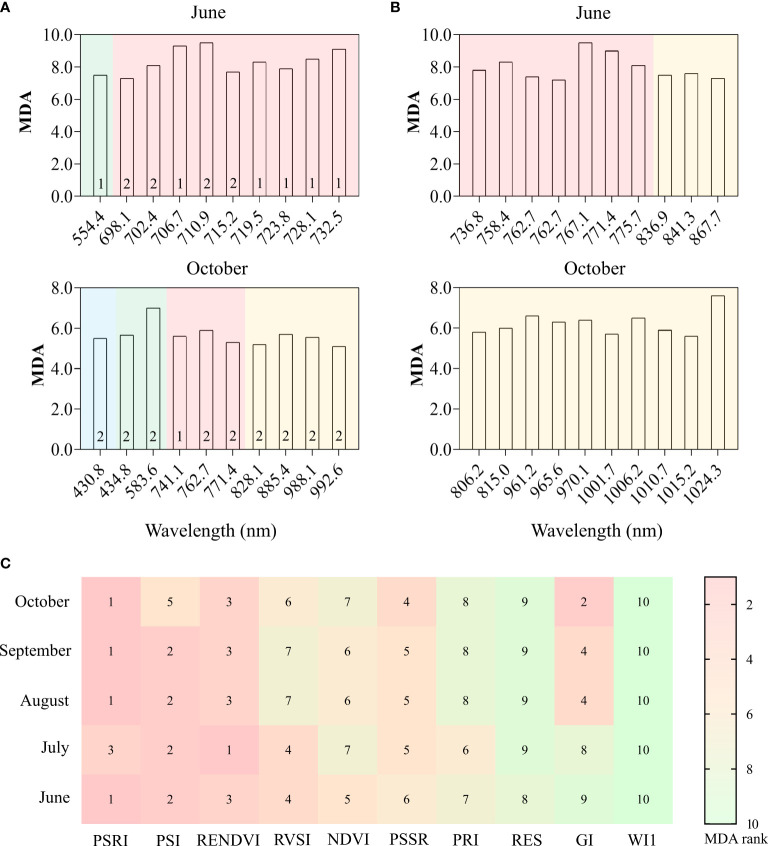
Mean Decrease Accuracy (MDA) of the top 10 variables ranking for all spectral features **(A)**, spectral wavelength **(B)**, and VIs **(C)** for separating the two groups of trees using the RF classification model. Numbers “1” and “2” in **(A)** indicate 1_st_ and 2_nd_ derivative, respectively. The different backgrounds in **(A, B)** indicate the band region: blue (blue), green (green), red edge (red), as well as NIR (yellow). The numbers in **(C)** represent the MDA rank of each variable.

Among all the features, the derivatives performed best in separating the two groups of trees, followed by spectral bands and VIs. The classification accuracy was much lower using only DN values from the RGB image than when using hyperspectral images from May to August, while it was only slightly lower in September and October (**Figure 10**). In the RF model using all features, the red edge bands showed higher importance than the other bands for identifying the two groups of trees in June (early stage of PWD infestation), while the NIR bands contributed more than other bands in October (late stage of PWD infestation) (**Figures 11A, B** **)**. For VIs, the red edge-based indices (e.g., PSRI, PSI and RENDVI) played a more important role in the RF model than others (e.g., NDVI, PSSR, PRI) (**Figure 11C**). This indicated a higher potential of the red edge bands for the early detection of PWD infestation.

## Discussion

### Optimal monitoring period of PWD

**Figure 12:**
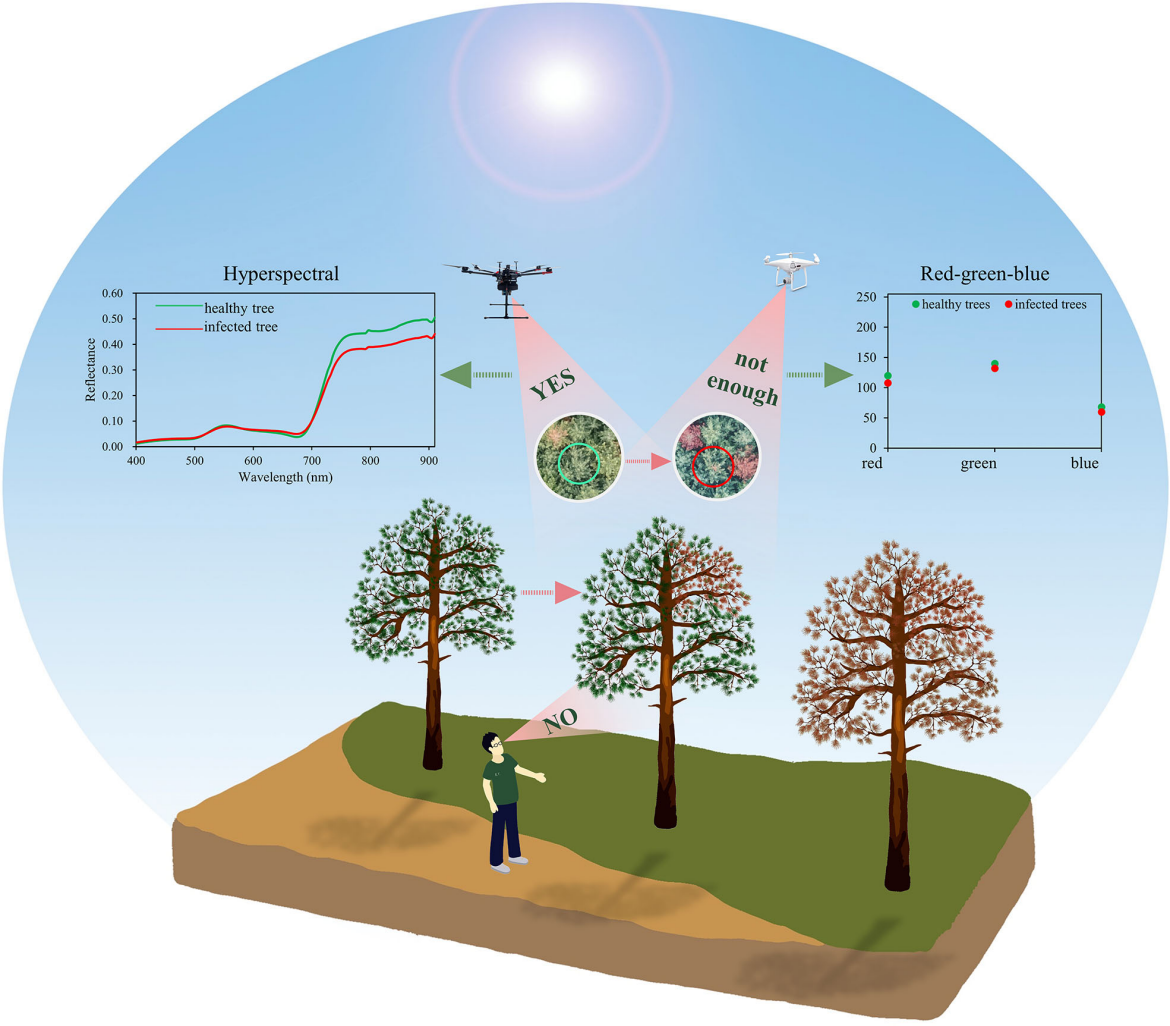
A schematic diagram of tree crown changes and the limited range of human observations.

In our study, variations in the UAV-based hyperspectral features due to PWD infestation were first noticeable in June, before changes in the RGB data and field investigation were visible. Multiple factors can explain this difference. On the one hand, changes always begin at the tree crown, and our ability to observe them from the ground is limited. On the other hand, the RGB data may not have enough information to succeed in early detection (**Figure 12**). Significant differences in spectral features first appeared in June, but the classification accuracy was not promising at this time. With higher separability, July seems to be the optimal monitoring period. Understandably, it is important to ensure that all infested trees are found before the end of the flight period of vector insects so that they can be removed later to prevent spread next year. However, the definition of the “optimal monitoring period” should be more comprehensive and take into consideration, at the same time, factors such as monitoring time, monitoring accuracy, the generation of pest, and the cost of taking control measures. Our results showed that the PWD monitoring accuracy of July was lower than that of October, even though the monitoring time was earlier. If we monitor pine trees and take control measures (e.g., felling) in June, many non-infested trees would also be impacted due to relatively low detection accuracy (e.g., non-infested trees were incorrectly identified as infested trees). Conversely, if pine trees are detected in October, more pine trees would be infested from June to October and the cost of treatment would be higher, even though the detection accuracy is higher at this time. In addition, the vector insects in our study are one generation per year, so the trees infested with PWN spread by vectors in flight periods will not cause new infestation later in a year. This indicates that it is enough to take control measures only once a year in the late stage of infestation (e.g., in October), instead of felling twice in the early stage and late stage of infestation (e.g., in June and October), because the vector insects only fly once a year and will not cause new spread within a year. However, if vector insects are two generations per year (e.g., *Ips typograhus*), then summer sanitation (e.g., in June) is necessary. Therefore, to describe the “optimal monitoring period” more accurately, multiple factors such as monitoring accuracy, time, the generation of pest and treatment cost needs to be considered in future research.

### Potential spectral bands for PWD early detection

In this study, a significant difference in the red edge region was first detected in June when this portion of the spectrum has stronger separability (**Figures 5B**, **11**), demonstrating great potential for PWD early detection. This was also confirmed by the fact that the most important features in the RF model and the most reactive VIs at the early stages of a PWD infestation were mainly red edge-based variables (**Figure 11**). These results are consistent with other studies of forest pests and diseases (Dennison et al., 2010; Fassnacht et al., 2014; Bárta et al., 2021; Einzmann et al., 2021; Huo et al., 2021; Zhou et al., 2021; Yu et al., 2021c; Bárta et al., 2022). In a PWD study, red edge indices from needles and UAV-based hyperspectral data produced the highest accuracy for *B. xylophilus* early detection (Yu et al., 2021d). Similar results have also been found with the red edge band derived from ground and satellite spectral data to monitor early-stage bark beetle damage at the needle, crown, and stand levels (Abdullah et al., 2019a; Abdullah et al., 2019b). In another study, red-edge based indices were proved to be more sensitive during the early infestation of *Ips typographus* (Bárta et al., 2022). Specifically, the red edge position shifted to the shorter wavelength with an increasing degree of infestation (also called “blue shift”). This “blue shift” is an indicator of chlorophyll and leaf area loss (Rock et al., 1988).

In addition to red edge region, other bands also shown their potential in forest health monitoring. The short-wave infrared (SWIR) region of the spectrum is also key for distinguishing healthy trees from those damaged by *Ips typographus* and *Dendroctonus rufipennis* (Foster et al., 2017; Abdullah et al., 2019b; Bárta et al., 2021; Huo et al., 2021). Other studies also found that healthy trees normally have higher reflectances than damaged trees in the near infrared (NIR) bands (Liu et al., 2021; Ortiz et al., 2013; Yu et al., 2021d). The spectral features of NIR and SWIR are related to the leaf cell structure and water content (Carter and Knapp, 2001; Entcheva Campbell et al., 2004; Einzmann et al., 2021). Therefore, in addition to the red edge bands, other regions, such as NIR and SWIR, also have potential in the early monitoring of PWD.

### Comparison of HSI and RGB data in early monitoring of PWD

Most studies used RGB data to detect PWD-infested trees with obvious discoloration (at the middle or late stage of a PWD infestation) instead of early-stage detection (Hu et al., 2020; Qin et al., 2021; Xia et al., 2021; Zhang et al., 2021). However, detection accuracy is not promising when using RGB data at early in a PWD infestation. Early detection of PWD using RGB data had an accuracy of only 0.465–0.508 (Wu et al., 2021) and the low performance was likely caused by insufficient spectral information in these data.

In this study, HSI data performed better than RGB images in separating infested and control trees at the early stage of the PWD infestation. From May to August, the classification accuracy of HSI was 0.07–0.16 higher than that of RGB, while that of HSI data was only 0.02–0.03 higher in September and October. The classification accuracy using RGB data can achieve 0.74–0.92 from August to October, but only 0.47–0.66 from May to July. The results indicate that only using RGB data can also successfully separate the two groups of trees at the late stage of a PWD infestation but cannot provide early detection of PWD.

To sum up, there is a trade-off between HSI and RGB data selection. HSI data perform better in PWD early detection but have a higher cost, more complex data processing and tighter requirements for suitable weather conditions. UAVs equipped with RGB cameras can rapidly collect high-resolution images of tree crowns with low cost, high flexibility, and low requirements for clear sky conditions. However, using only RGB data cannot be used for early PWD detection. According to previous studies (Abdullah et al., 2019b; Bárta et al., 2021; Liu et al., 2021; Ortiz et al., 2013; Yu et al., 2021d), the red edge, NIR, and SWIR regions were considered as sensitive bands for early monitoring. For example, some studies found that healthy trees normally have higher reflectances than damaged trees in the NIR regions (Ortiz et al., 2013; Liu et al., 2021; Yu et al., 2021d). Based on this spectral information, replacing the RGB camera with an equally simple NIR instrument would likely considerably improve infestation detection without hyperspectral data. Another effective method is to design a multispectral camera with bands selected from HSI data dedicated to early monitoring of PWD, which will be the goal of our next studies.

## Conclusion

The UAV-based RS data used in our study successfully detected changes in the spectral behavior of PWD-infested trees. Sample trees were monitored from May to October. In June, before changes were noticeable in the RGB data and field investigation, changes in spectral features were first detected in the hyperspectral data.

Several spectral features were employed to detect variations in the spectrum reaction of infested trees compared to non-infested trees. The spectral reflectance of infested trees was altered in the visible, red edge and NIR bands. The most discriminative features for separating the two groups’ trees were the derivatives and the spectral reflectance, over the REPs or VIs. By using the RF algorithm, the two groups were successfully separated from July to October.

## Data availability statement

The original contributions presented in the study are included in the article/supplementary materials. Further inquiries can be directed to the corresponding author.

## Author contributions

RY and LH designed the methodology, analyzed the data, and wrote the manuscript. HH, YQL, YJL, LFY, and LR reviewed the manuscript. RY, HL, and LYY conducted the field surveys RY, BG, YY, and LR completed the chart making. All authors contributed to the article and approved the submitted version.

## Funding

This work was funded by the “National Key Research & Development Program of China (2021YFD1400900)”, “Major emergency science and Technology Project of National Forestry and Grassland Administration (ZD202001)”.

## Acknowledgments

We gratefully acknowledge Xin Luo and Sixun Ge for help with producing **Figure 4**. We would like to thank IRIS Inc. (Beijing, China) for the UAV-based hyperspectral image data collection and data preprocessing.

## Conflict of interest

The authors declare that the research was conducted in the absence of any commercial or financial relationships that could be construed as a potential conflict of interest.

## Publisher’s note

All claims expressed in this article are solely those of the authors and do not necessarily represent those of their affiliated organizations, or those of the publisher, the editors and the reviewers. Any product that may be evaluated in this article, or claim that may be made by its manufacturer, is not guaranteed or endorsed by the publisher.
